# Persona of Transition Metal Ions in Solids: A Statistical Learning on Local Structures of Transition Metal Oxides

**DOI:** 10.1002/advs.202202756

**Published:** 2022-07-24

**Authors:** Huaxian Jia, Matthew Horton, Yanan Wang, Shengjie Zhang, Kristin A. Persson, Sheng Meng, Miao Liu

**Affiliations:** ^1^ Beijing National Laboratory for Condensed Matter Physics and Institute of Physics Chinese Academy of Sciences Beijing 100190 China; ^2^ School of Physical Sciences University of Chinese Academy of Sciences Beijing 100049 China; ^3^ Songshan Lake Materials Laboratory Dongguan Guangdong 523808 China; ^4^ Materials Science Division Lawrence Berkeley National Laboratory Berkeley CA 94720 USA; ^5^ Molecular Foundry Lawrence Berkeley National Laboratory Berkeley CA 94720 USA; ^6^ Department of Materials Science and Engineering University of California Berkeley Berkeley CA 94720 USA; ^7^ Center of Materials Science and Optoelectronics Engineering University of Chinese Academy of Sciences Beijing 100049 China

**Keywords:** ionic radius, Jahn–Teller effect, magnetism, statistics, transition metal oxides

## Abstract

The local structure of a transition metal (TM) ion is a function of cation elements and valence states. More than that, in this work, by employing a trove of first‐principles data of TM oxides, the local structures of TM cations are statistically analyzed to extract detailed information about cation site preference, bond length, site structural distortion, and cation magnetization. It is found that cation radius alone poorly describes the local structure of a transition metal oxide, while the statistics of coordination number as well as the TM—O bond length distribution, especially that of the 3d TMs, can provide comprehensive knowledge for understanding the behavior of TM elements. Based on these statistics, the interplay of site distortion due to the Jahn–Teller effect, cation site similarity, and a new set of ionic radii are all obtained to chart the “persona” of transition metal ions in solids.

## Graphical abstract

Harnessing a trove of high‐quality computational data from the Materials Project, this paper statistically extracts the detailed “shape” and “size” of cations in the ionic compounds as a function of valence states and magnetization. Cation site similarity and a new set of ionic radii are also obtained to chart the “persona” of transition metal ions in solids.

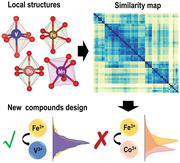

## 1 Introduction

The sizes of the atoms and their site preferences in a solid are important aspects of the elements. It was realized by Dalton that the atoms can be categorized into particular element species without knowing their radii. Wyckoff found that each element should have a finite atomic size to rationalize the crystallographic data from X‐ray measurements.^[^ ^1^ ^]^ The Bohr model of atomic structure, published in 1913, suggested that the size of an atom should be species‐dependent. Later, Goldschmidt and Pauling proposed the concept of “ionic radius” independently.^[^ ^2^ ^]^ Pauling even developed the concept of “resonated metallic valence” to understand the variation of atom radius in alloys, whose size is also a function of electronic structure.^[^ ^3^ ^]^ For ionic compounds, it gets even more complicated because of the introduction of valence states. At present, the most widely used ionic radius values are a set of effective ion radius data based on oxidation states, coordination numbers, and spin states, published by Shannon and Prewitt in the 1970s, derived from a statistical analysis of existing crystallographic data^[^ ^4^ ^]^ available at that time. Harnessing the large trove of crystallographic data now available, which is several times larger than that in the 1970s, the ionic size definition can be revisited in a more comprehensive fashion.

Shannon's ionic radii are obtained from ≈700 experimental crystal structures in the 1970s.^[^ ^4^ ^]^ Because of the insufficient data, the ionic radius is mostly an average over a few data points, therefore the value of ionic radii may not be able to capture the correct statistical trends of bond‐length distribution over the vast material phase space. More importantly, some useful information might be overlooked by not surveying enough data. For example, what is the bond‐length distribution of ions in solids? Are the octahedral and tetrahedral sites typically geometrically distorted? And, to what extent are they distorted? Currently, the size of the experimental dataset, accumulated in the past several decades by crystallographic scientists worldwide, exceeds ≈200 thousand crystal structures (e.g., ICSD), ≈10 times larger than that of Shannon's age.^[^ ^5^ ^]^ However, there are only ≈60 000 nonidentical crystal structures after removing duplicated structures and disordered structures.^[^ ^6^ ^]^ Therefore, the latest statistical studies on the local bond‐length distribution of alkali and alkaline earth metals,^[^ ^7^ ^]^ metalloids, post‐transition metals,^[^ ^8^ ^]^ TMs,^[^ ^9^ ^]^ and the study of coordination environment,^[^ ^10^ ^]^ have been carried out over a small data size (≈100 structures per cationic species): too few to extract a high‐resolution distribution of bond lengths and atomic size variations.

Fortunately, the recent advances of high‐throughput first‐principles calculations (e.g., Materials Project, OQMD, and atomly.net, etc.) boost the data size of inorganic crystals to an even larger scale (≈140 000–1 000 000 inorganic crystal structures), well exceeding the size of experimental datasets.^[^ ^11^ ^]^ In addition, those databases provide additional information of inorganic compounds, such as the formation energy, magnetization, as well as the high‐resolution crystal structures, covering a vast phase space of inorganic crystalline structures. Hence, it is worthwhile to revisit this classical topic of ionic radius, as the availability of abundant data brings new knowledge of the local structures of materials at the atomic level.

This work statistically studies the local structures of the TM oxides, especially the 3d metal oxides, by employing the computational data of ≈120 thousand inorganic compounds from Materials Project (MP).^[^ ^11a^ ^]^ We obtain a comprehensive picture of the local structures of TM cations, such as the site preference, cation sizes, and site symmetry distortion, as a function of valence and magnetic states. Although the crystal structures from the density functional theory (DFT) at the PBE+U level^[^ ^12^ ^]^ usually overestimate the lattice parameters by 1–2% and sometimes 3% (Table S1, Supporting Information) compared to the experimental values, the calculated structures can capture the overall trend of the cation size distributions as well as the structure distortion in a fairly accurate manner.^[^ ^13^ ^]^ It is found that the TM cations generally prefer 6‐coordinated octahedral sites, unless there is a large ionic potential. The ionic sizes of 3d TM metals are generally in good agreement with Shannon's ionic radii, however, the statistic distribution of the bond lengths is a function of the valence states, compound stability, and magnetization of cations, hence it provides a unique and detailed persona to describe the local structures of cations. Each cation has its own personality, for example, Cr^3+^ prefers octahedral site with a small span of bond lengths, while the V^5+^ can fit into either octahedral or tetrahedral sites with a large span of bond lengths. The statistical analysis also demonstrates the spin states and the Jahn–Teller effect of TMs in solids, which manifest the orbital‐related electronic structures of compounds. Knowledge of the local structural similarity can then be clearly extracted from the data, hence helping us understand the atomistic mechanisms (e.g., the intercalation phenomena in energy materials) and guide the rational design of materials (e.g., via species substitution).

## 2 Results

**Figure 1:**
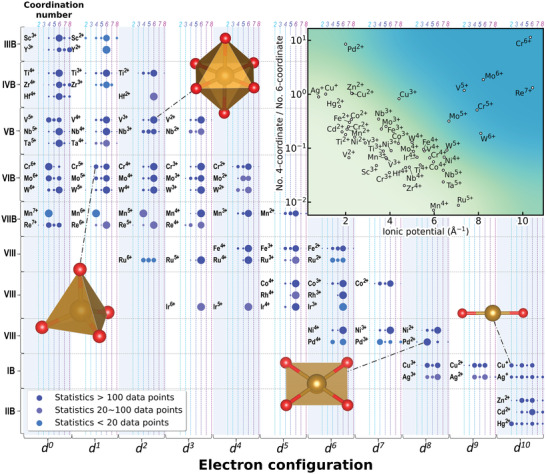
The site preferences for transition metal (TM) cations in solids. According to the statistics of the 54 379 distinctive TM sites in 32 055 compounds, the coordination numbers of the 25 TMs are extracted/sorted as a function of their valence states (number of d orbitals). In the figure, the likelihood of the coordination number (range from 2 to 8) of a TM cation to exist is shown as the area of the corresponding circles. The inset presents the ratio of the 4 to 6 coordination versus ionic potential of each cation species, and the octahedral‐site‐preferred cations generally appear on the bottom‐left side. The deeper the color indicates the stronger the second‐order Jahn–Teller effect.

The TM cations have preferred “shapes” in oxides. **Figure** **1** presents the statistical coordination numbers of TM cations surrounded by oxygen anions, which reveals the site preference of 3d, 4d, and 5d TM cations (except for the Tc, Os, Pt and Au as the data for those compounds are fairly limited). As shown in Figure 1, it is found that most of the TMs favor octahedral environments over the tetrahedral environment in oxides. The V^5+^, Cr^6+^, Mo^6+^ and Re^7+^ are exceptions, as they tend to form the tetrahedral environment not only because they all have a small radius to fit into the smaller space in tetrahedral sites, but more importantly, the presence of the second‐order Jahn–Teller (SOJT) effect in d^0^ and d^1^ TM cations tends to destabilize and deform the octahedral sites.^[^ ^14^ ^]^ Some cations, such as the Ni^4+^ and Co^4+^, have equal or even smaller radius compared to that of V^5+^, but still lean toward forming the octahedral structures when SOJT is weak. Overall, SOJT‐active cations are likely to stay in an off‐centered low‐symmetric environment. Pd^2+^ favors the 4‐coordinated planar site, which can accommodate the larger‐size cations.

It also can be observed that the site preference is a function of ionic potential, which is defined as the ratio of the electrical charge (*Z*) to the Shannon's ionic radius (*r*) of an ion, *φ* = *Z*/*r*. As shown in the inset of Figure 1, the tetrahedral coordination‐preferred cations are all located in the upper right corner of the plot, while the octahedral‐coordination‐preferred ones stay in the lower left. This, to some extent, violates the classical Pauling rule in solid‐state chemistry, which states that the smaller central cation tends to exhibit low‐coordination numbers., E.g., the size of Cr^3+^ (75.5 pm) is smaller than Zn^2+^ (88 pm), but Cr^3+^ prefers the octahedral site, while Zn^2+^ is more likely to reside in the tetrahedral site. Overall, the ions in solids should not be described as hard spheres with a fixed radius but, rather, the polarization of ions should be taken into account to be able to determine their site preference.^[^ ^14^, ^15^ ^]^

**Figure 2:**
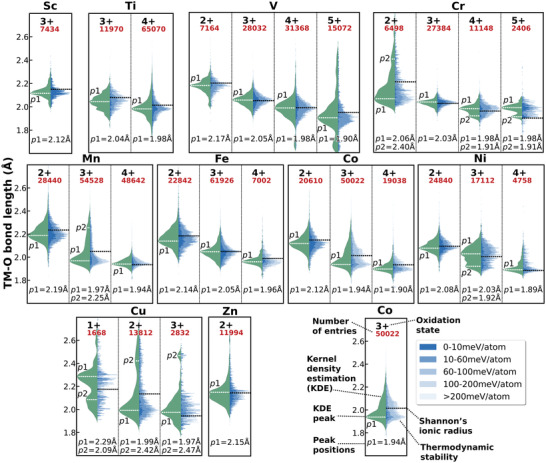
The distribution of 3d TM—O bond length. In each subplot, the right side presents the distribution of the TM—O bonds, and the left side represents the normalized “canonical” TM—O bonds according to the thermodynamic stability. The black dashed lines mark the TM—O bond length from the Shannon radius, and the white dashed lines demonstrate the TM—O bond lengths of this work, which normally differ from the Shannon's value by up to ≈3%, sometimes even larger (such as Cu^3+^ and Cr^5+^), depending on the cation.

The TM—O bond lengths are very important features for TM oxides. Since octahedral coordination is preferred for most cations, **Figure** **2** demonstrates the distribution of 3d TM—O bond lengths for octahedral TM sites, extracted from a computational dataset of 100 944 distinctive TM sites from 19 341compounds, in which 15 719 octahedral sites in 3251 structures are originally from the ICSD. All structures were fully relaxed by first‐principles calculations, and the statistical results of the TM—O bond‐length distributions are obtained by taking all the O atoms in the first shell into the count. Previously, Shannon et al. employed the average bond lengths of the six bonds in each octahedral site, which smoothed out the distortion of the octahedron^[^ ^4^ ^]^ limited by the small dataset he had at that time. Taking the advantage of the high‐quality materials database as of today, here, we are able to take into account the anisotropy of octahedral site, the thermodynamic stability and the magnetization of compounds. In Figure 2, the shades of the color denote the thermodynamic stability (energy above the hull) of each compound, and the “canonical” bond‐length statistics on the left is normalized by the Maxwell–Boltzmann distribution according to the thermodynamic stability of each compound, indicating that the bond lengths are weighted toward more thermodynamically stable compounds to prevent unstable, theoretical compounds from biasing our conclusions. The ionic radius from Shannon's work is marked as a black dashed line.

**Figure 3:**
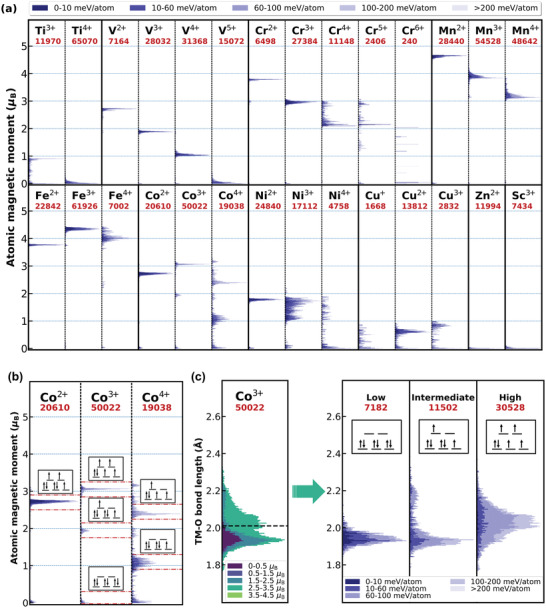
a) When TM cation in octahedral sites, the distribution of the atomic magnetic moments of the 3d TMs as a function of oxidation states. b) For example, the spin states of cobalt cations vary with their valence states. c) Based on the magnetization, the Co^3+^—O bond length distribution is decoupled into the low‐, intermediate‐, and high‐spin states to manifest the Jahn–Teller effect.

As shown in Figure 2, the overall trend of bond lengths and their dependency versus the cation valence primarily agree well with the work of Shannon,^[^ ^4b^ ^]^ however, the values of the bond lengths are fairly different. Since the TM—O bond length of cations falls into a range and each cation has its signature distribution, the previous use of a single value to define the ionic radius is less informative. In most cases, the bond length of the cation falls within a 0.2–0.3 Å range, and some cations, such as V^5+^, Cr^2+^, Mn^3+^, Co^3+^, Cu^+^, Cu^2+^, and Cu^3+^, exhibit a wide distribution of the bond lengths, up to 0.4–0.8 Å. For example, the TM—O bond length of Mn^3+^ spans from 1.91 to 2.35 Å, with at least two peaks at 1.97 and 2.26 Å. Those wide‐span or multiple TM—O lengths have a clear connection with the cation magnetization (**Figure** **3**a), as the 3d electrons of those cations can be modulated into multiple spin‐polarization states, e.g., the TM—O length of Ni^3+^ (magnetic state is around 1–2 *μ*_B_) spreads over a wide range from 1.89 to 2.23 Å, because Ni^3+^ is Jahn–Teller (JT) active. The Co^3+^ is also JT active, and its bond length can be decomposed into the low, intermediate, and high spin states as shown in Figure 3c. Under octahedral crystal field, the low spin (LS) state corresponds to a high‐symmetry configuration owning to its highly degenerated t_2g_ orbitals, whereas the intermediate spin state demonstrates the JT effect as the 1/3 of the bonds are elongated. The high spin Co^3+^ state is energetically less favorable due to spin polarization, with most of the compounds having an elevated energy above hull (≈60–100 meV per atom), signifying that the energy needed to populate the electron from t_2g_ to e_g_ is ≈ 30–50 meV/*μ*_B_. Similarly, the JT effect can also be statistically identified in the Cr^2+^, Mn^3+^, Cu^2+^ and Cu^3+^ (LS) cations (low/high spin state of Cu^3+^ are shown in Figure S1, Supporting Information).

**Figure 4:**
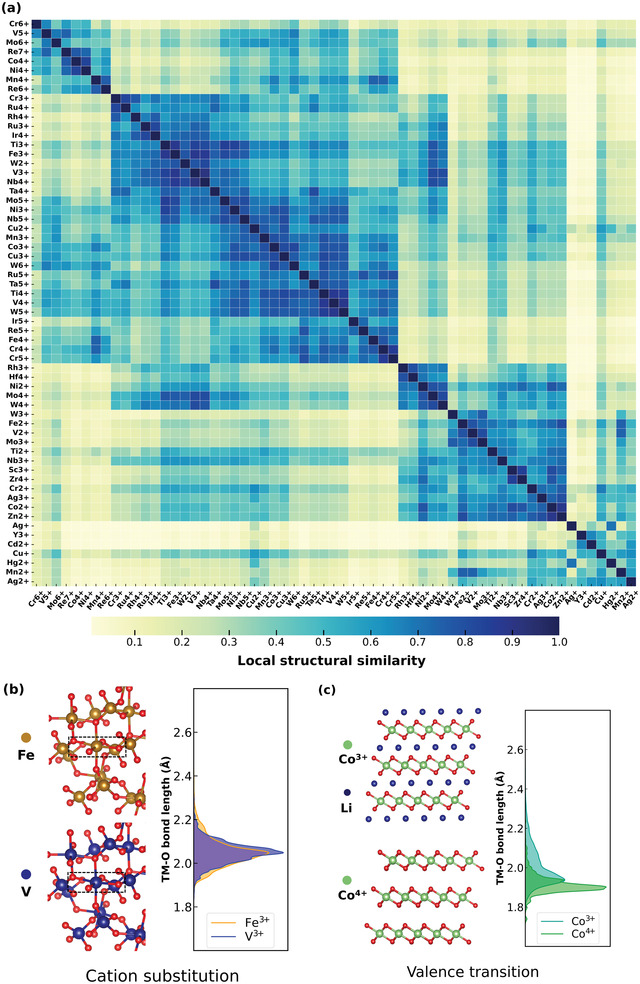
a) The similarity map of transition metals obtained from hierarchical clustering machine learning. Dark color denotes the elevated similarity, vice versa. b) The Fe^3+^ and V^3+^ share a very similar bond length distribution curve, hence it is likely that cation substitution is possible between them. c) There is a large overlap between the Co^3+^ and Co^4+^ bond length distributions, as such the valence transition tends not to lead to significant structural distortions or phase change.

The local structure similarity can be reflected from the overlap area of the “canonical” bond length distribution. For example, the bond lengths distribution of Fe^3+^ and V^3+^ have a very large intersection, hence the geometry of Fe^3+^ site should be able to accommodate the V^3+^ comfortably, as shown in **Figure** **4**b. Extending the same treatment to all 3d TM cations, Figure 4a is obtained as a “map” to chart the similarity of cations. Essentially, it represents the likelihood for cations to substitute each other in compounds. Those cations are hierarchically clustered via machine learning and ranked according to their local structure similarity, so that the most similar cations are placed next to each other on the plot. In general Figure 4a can serve as a synthetic guidance for justifying the chance of cation substitution or doping, e.g., Fe^3+^ and V^3+^ have a similar local environment and the same valence state, hence, it is feasible to discover new vanadium oxide materials from isostructural iron oxides.^[^ ^16^ ^]^ It is noticeable that Figure 4a is similar but differs from the Figure S2 (Supporting Information), which is a similarity diagram of cations only based on the ionic radius. It is suggested that the radius of cations alone is unlikely to address the site preference precisely. The similarity map based on the Jensen‐Shannon divergence, as charted in Figure S3 (Supporting Information), is in line with Figure 4a. Previously, similar work was done using a much smaller dataset for several cations (only 7 TM cations),^[^ ^17^ ^]^ and this work is conducted with a much larger dataset to chart the similarity for all TM cations.

Figure 4a can also be used to rationalize the structural changes of cations during chemical reactions in some cases. As shown in Figure 4c, the local environment of the Co cation changes only slightly when transiting from the 3+ state to 4+ state during lithiation, therefore Co‐containing compounds often serve as effective Li intercalation hosts, whereas Mn tends to have large structural change when transitioning from 2+ state to 3+. This explains why it is generally challenging for a TM to reversibly change its valence state more than one step (e.g., from 2+ to 4+) in intercalating battery cathodes as it usually requires a significant change in cation local structure. It also tells us that the Ni, Mn, and Co are mixable to form disordered Nickel‐Manganese‐Cobalt (NMC) cathode as the Ni, Mn, and Co sites share a similar local environment during the electrochemical reactions.^[^ ^18^ ^]^ It hints that Cr can be incorporated into disordered rock salt cathode materials with Mo^[^ ^19^ ^]^ and Mn,^[^ ^20^ ^]^ according to the similarity of cations in their respective local structure during redox reactions.

## 3 Discussion

The degree of dispersion in distributions of TM—O bonds is an important character of TM cations, which cannot be described through the constant values of the ionic radius. The dispersion level of bond lengths represents the degree of symmetry of the cations. Cr^3+^ (3d^3^), Mn^4+^ (3d^3^) and Co^3+^ (3d^6^, low spin) are the types of cations that prefer highly symmetric octahedral environment with oxygen anions, while the d^0^ metals such as the Y^3+^, V^5+^, and Mo^6+^ like low symmetry octahedral sites.^[^ ^21^ ^]^ Cr^2+^, Mn^3+^, Co^3+^ (intermediate spin), Ni^3+^, Cu^2+^, Cu^3+^ cations can manifest a strong Jahn–Teller effect, therefore these cations are more comfortable staying at a low‐symmetry and strongly distorted octahedral environment.

It is often possible to see multiple peaks for the TM—O bond length, especially when the octahedrons are distorted, and since these distortions can be very strong., E.g., Co^3+^ has two bond length peaks due to their magnetic states. As shown in Figure 3a, there is an interplay between magnetic moment, electronic orbital configuration, and the geometric structure, hence it is feasible to further split the cation local structures into sub‐groups according to their spin state, which cannot be done within the framework of Bond Valence method.^[^ ^14c^ ^]^ Cu^3+^ and Cr^5+^ have two bond length peaks, and Ni^3+^ has more than two bond length peaks, as they have multiple spin states too.

The data employed in this work is all from the first‐principles calculations, which is often systematically 1–2% (sometimes up to ≈3%) off compared to the experimental values (Table S1, Supporting Information), but they are highly comparable among the theoretical data provided that they are calculated by the same method. The calculated crystal structure has an even higher resolution up to 0.01 Å, when the magnetism and subtle lattice distortion are captured at the atomistic level, and with thermal expansions eliminated. It is found that when many calculated TM—O bond lengths are off by more than 2% compared to the experimental value, those experimental values (such as V^2+^, and Cu^3+^) may need to be updated.^[^ ^4b^ ^]^ Based on the calculated data, a table of cation radii distribution is summarized as a supplement to Shannon's ionic radii (**Table** **1**). The table contains the trends and distributions of the local structure of TM cations, and as such the “persona” of transition metal ions in solids can be clearly revealed.

**Table 1:** The list of ionic radius and bond‐length distributions for *3d* transition metals

Cation species	Pool size for statistics	MP radius [Å]	Shannon radius [Å]	MP vs Shannon radius (%) [Å]	Jahn–Teller activity	Bond length of Peak1 (FWHM) [Å]	Bond length of Peak2 (FWHM) [Å]	Peak2 to Peak1 height ratio	Mean bond length ± SD [Å]
Sc^3+^	7.4k	0.870	0.885	−0.015 (−1.7%)		2.116 (0.048)			2.130 ± 0.061
Ti^3+^	12.0k	0.792	0.81	−0.018 (−2.2%)		2.040 (0.117)			2.052 ± 0.072
Ti^4+^	65.1k	0.730	0.745	−0.015 (−2.0%)		1.977 (0.066)			1.990 ± 0.091
V^2+^	7.2k	0.909	0.93	−0.021 (−2.3%)		2.174 (0.051)			2.169 ± 0.076
V^3+^	28.0k	0.790	0.78	+0.010 (+1.3%)		2.050 (0.069)			2.050 ± 0.062
V^4+^	31.4k	0.715	0.72	−0.005 (−0.7%)		1.983 (0.125)			1.975 ± 0.099
V^5+^	15.1k	0.661	0.68	−0.019 (−2.8%)		1.899 (0.119)			1.921 ± 0.149
Cr^2+^	6.5k	0.918	0.94	−0.022 (−2.3%)	√	2.060 (0.144)	2.401 (0.039)	0.20	2.178 ± 0.160
Cr^3+^	27.4k	0.766	0.755	+0.011 (+1.5%)		2.032 (0.038)			2.026 ± 0.049
Cr^4+^	11.1k	0.710	0.69	+0.020 (+2.9%)		1.975 (0.084)	1.913 (0.010)	0.54	1.970 ± 0.059
Cr^5+^	2.4k	0.691	0.63	+0.061 (+9.7%)		1.984 (0.046)	1.906 (0.018)	0.54	1.951 ± 0.084
Mn^2+^	28.4k	0.951	0.97	−0.019 (−2.0%)		2.188 (0.121)			2.211 ± 0.103
Mn^3+^	54.5k	0.782	0.785	−0.003 (+0.4%)	√	1.969 (0.085)	2.255 (0.017)	0.17	2.042 ± 0.120
Mn^4+^	48.6k	0.695	0.67	+0.025 (+3.7%)		1.941 (0.046)			1.955 ± 0.053
Fe^2+^	22.8k	0.910	0.92	−0.010 (−1.1%)		2.140 (0.125)			2.170 ± 0.101
Fe^3+^	61.9k	0.788	0.785	+0.003 (+0.4%)		2.048 (0.071)			2.048 ± 0.076
Fe^4+^	7.0k	0.717	0.725	−0.008 (−1.1%)		1.961 (0.044)			1.977 ± 0.068
Co^2+^	20.6k	0.865	0.885	−0.020 (−2.3%)		2.120 (0.089)			2.125 ± 0.096
Co^3+^(LS)	7.2k	0.689	0.685	+0.004 (+0.6%)		1.933 (0.048)			1.949 ± 0.037
Co^3+^(IS)	11.5k	0.735			√	1.935 (0.069)	2.119 (0.019)	0.17	1.995 ± 0.109
Co^3+^(HS)	30.5k	0.774	0.75	+0.024 (+3.2%)		2.025 (0.153)			2.034 ± 0.090
Co^4+^	19.0k	0.666	0.67	−0.004 (−0.6%)		1.898 (0.043)			1.926 ± 0.079
Ni^2+^	24.8k	0.837	0.83	+0.007 (+0.8%)		2.078 (0.081)			2.097 ± 0.076
Ni^3+^	17.1k	0.764	0.74	+0.024 (+3.2%)	√	2.029 (0.181)	1.983 (0.010)	0.82	2.024 ± 0.087
Ni^4+^	4.8k	0.688	0.62	+0.068 (+11.0%)		1.891 (0.032)			1.948 ± 0.082
Cu^+^	1.7k	0.966	0.91	+0.056 (+6.2%)		2.286 (0.107)	2.086 (0.100)	0.57	2.226 ± 0.172
Cu^2+^	13.8k	0.873	0.87	+0.003 (+0.3%)	√	1.992 (0.123)	2.421 (0.138)	0.21	2.133 ± 0.201
Cu^3+^(LS)	1.5k	0.764	0.68	+0.084 (+12.4%)	√	1.954 (0.130)	2.468 (0.061)	0.29	2.024 ± 0.186
Cu^3+^(HS)	1.3k	0.783				1.991 (0.081)			2.043 ± 0.076
Zn^2+^	12.0k	0.905	0.88	+0.025 (+2.8%)		2.150 (0.124)			2.165 ± 0.136

The cation similarity shown in Figure 4a may significantly expand the phase space of the inorganic compounds. Currently, there are 88k known TM‐containing compounds in Materials Project, 27k of which are from ICSD. Employing those compounds as the starting structures, we have generated another ≈290k new structures, and ranking them with the site similarity “map” as shown in Figure 4a. After the calculation of the top 62k compounds, we found that 12k new compounds fall into the thermostability region with a small energy above hull of 50 meV per atom or less, whereas 5k new compounds have an energy above hull even smaller than 20 meV per atom. These new lists of compounds can be found from the atomly.net materials database.^[^ ^11c^ ^]^ Likely, there is a large amount of phase space of possible inorganic compounds out there awaiting discovery.

## 4 Conclusion

By harnessing the recent advance of the high‐quality computational data of inorganic compounds, it is now feasible to extract the local structures of TMs in those compounds in a rigorous and comprehensive manner. In addition to the ionic radius obtained by pioneering scientists like Shannon, we are now able to extract the detailed local structures of TM cations, including the TM—O bond distribution and local magnetic moments. Hence, the “persona” of the TMs in oxides, and especially their site preference, cation size and site symmetry distortion as a function of valence and magnetic states, are statistically obtained in this work. The local structure similarity obtained thereafter has profound implications for designing new materials. Along with the availability of more high‐quality data, a new paradigm of materials science is around the corner to help us understand the material world in a more precise manner.

## 5 Experimental Section

A total of 12 0612 different inorganic crystal structures from the Materials Project database version 2019.05 were used in this study.

### Determination of Ionic Crystals

Compounds containing both metallic and strongly electronegative elements (N, O, S, F, Cl, Br, I, P, Se) as ionic crystals were considered, and a total of 81 278 ionic crystal structures were obtained. The local structures of the crystals were then analyzed by using the bond valence method^[^ ^22^ ^]^ in pymatgen^[^ ^23^ ^]^ to determine the valence of each atom. After the removal of 14 889 structures with nonassignable valence states, 66 389 structures remained.

### Determination of Coordination Numbers

The CrystalNN algorithm was used to determine the coordination number of cations in crystals.^[^ ^24^ ^]^ To distinguish the square planar motif from tetrahedral motif in some four‐coordinated cations, e.g. Pd^2+^, ChemEnv was used to provide a detailed description of the coordination information.^[^ ^25^ ^]^ The detailed statistics of all the four‐coordination cations are shown in Figure S4 (Supporting Information). All implementations above are available in the pymatgen library.

### Statistical Analysis

Oxygen coordination information was counted for all transition metal ion crystals. In each structure, different valence states of the same transition metal combined with different coordination numbers contributed a statistical sample (e.g., Fe3+:6, Fe4+:4…). To eliminate the influence of unstable structures on the coordination statistics results, structures larger than 300 meV per atom were not considered in the statistics.

The bond lengths in each structure were calculated using pymatgen, each M—O bond length in the cell was considered as a sample. When counting the bond length distribution, for each bond length sample, a weighting factor was introduced:

(1) $$w\;=\;\frac{1}{n/c}$$

to avoid duplication introduced by cell symmetry (*n* represents the total number of bonds of a certain transition metal ion boanalyzed in a sample of compounds, and *c* represents the number of coordination sites for that ion; in this paper, *c* = 6). The bond length in each crystal structure corresponds to a unique Δ*E*_hull_, and thermodynamically unstable structures with a Δ*E*_hull_ greater than 300 meV per atom were not counted.

Normalized “canonical” TM—O bond length distribution was obtained by Gaussian kernel density estimation function in the SciPy package,^[^ ^26^ ^]^ and a bandwidth of 0.15 Å was chosen for all cations.

For statistical analysis of bond lengths with magnetic moments, the weighting factor for the bond length statistics was determined in the same way as above, and the bond length in each O‐octahedron of the crystal structure is associated with a unique magnetic moment.

Python module statsmodels^[^ ^27^ ^]^ was used to calculate the weighted mean and standard deviation of bond lengths. Peak locations and peak widths were determined by SciPy, full width at half maximum (FWHM) was used, which was the width at the relative height of 0.5.

## Conflict of Interest

The authors declare no conflict of interest.

## Supporting information

Supporting Information

## Data Availability

The data that support the findings of this study are openly available in materials project at https://doi.org/10.1063/1.4812323, reference number 14.
