# Supplementary material for: Persona of Transition Metal Ions in Solids: A Statistical Learning on Local Structures of Transition Metal Oxides
Source: Adv Sci (Weinh). 2022 Jul 24;9(27):2202756. doi: 10.1002/advs.202202756 (PMC9507351; doi:10.1002/advs.202202756)
Supplement: Supplementary file 1 — Supporting Information [file ADVS-9-2202756-s001.pdf]

ADVANCED SCIENCE
Open Access

# Supporting Information

for *Adv. Sci.*, DOI 10.1002/advs.202202756

Persona of Transition Metal Ions in Solids: A Statistical Learning on Local Structures of Transition Metal Oxides

*Huaxian Jia, Matthew Horton, Yanan Wang, Shengjie Zhang, Kristin A. Persson*, Sheng Meng* and Miao Liu**

Supporting Information

# Persona of transition metal ions in solids: a statistical learning on local structures of transition metal oxides

*Huaxian Jia[1, 2, 3], Matthew Horton[4], Yanan Wang[1, 3], Shengjie Zhang[1,2], Kristin A. Persson[5, 6*], Sheng Meng[1, 2, 3*], Miao Liu[1, 3, 7*]*

[1]Beijing National Laboratory for Condensed Matter Physics and Institute of Physics, Chinese Academy of Sciences, Beijing 100190, China

[2]School of Physical Sciences, University of Chinese Academy of Sciences, Beijing 100049, China

[3]Songshan Lake Materials Laboratory, Dongguan, Guangdong 523808, China

[4]Materials Science Division, Lawrence Berkeley National Laboratory, Berkeley, California 94720, USA

[5]Molecular Foundry, Lawrence Berkeley National Laboratory, Berkeley, California 94720, USA

[6]Department of Materials Science and Engineering, University of California Berkeley, Berkeley, California 94720, USA

[7]Center of Materials Science and Optoelectronics Engineering, University of Chinese Academy of Sciences, Beijing 100049, China

*Corresponding author: mliu@iphy.ac.cn, smeng@iphy.ac.cn, kapersson@lbl.gov

**This PDF file includes:**

Figures. S1 to S4
Table S1
References

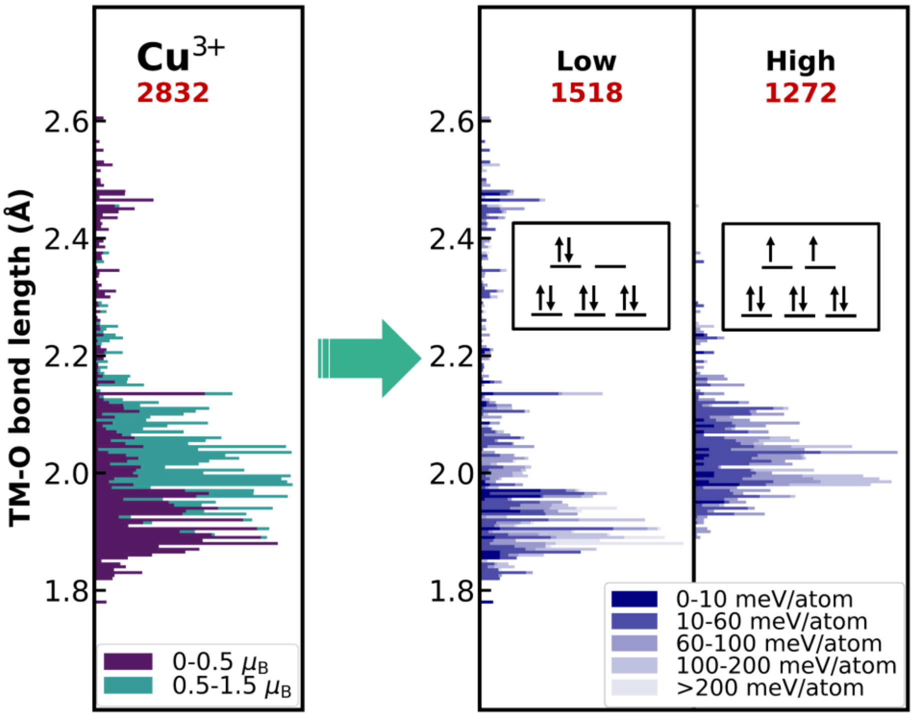


**Figure S1. $Cu^{3+}$-O bond length distribution as a function of the $Cu^{3+}$ magnetization.** The $Cu^{3+}$ cation at low- and high-spin states behaves differently and Jahn-Teller effect can be clearly manifested from the low-spin states.

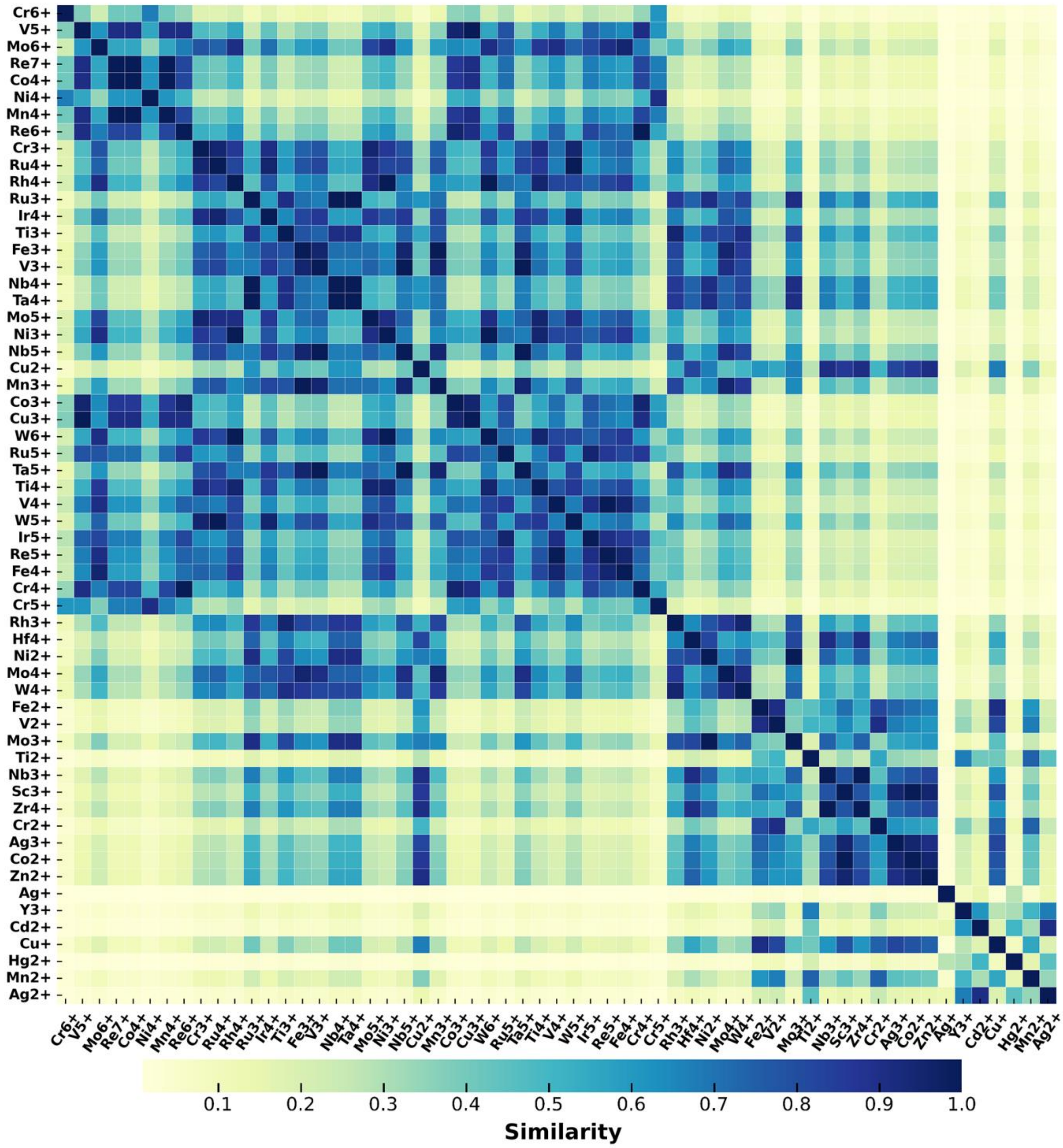


**Figure S2. The similarity of transition metal cations that derived from Shannon's ionic radius.** This figure adopts the same methodology as employed in Figure 4a to ensure that the two figures are comparable. And the figure essentially tells the likelihood of the cation substitution for transition metal ions in solid compounds.

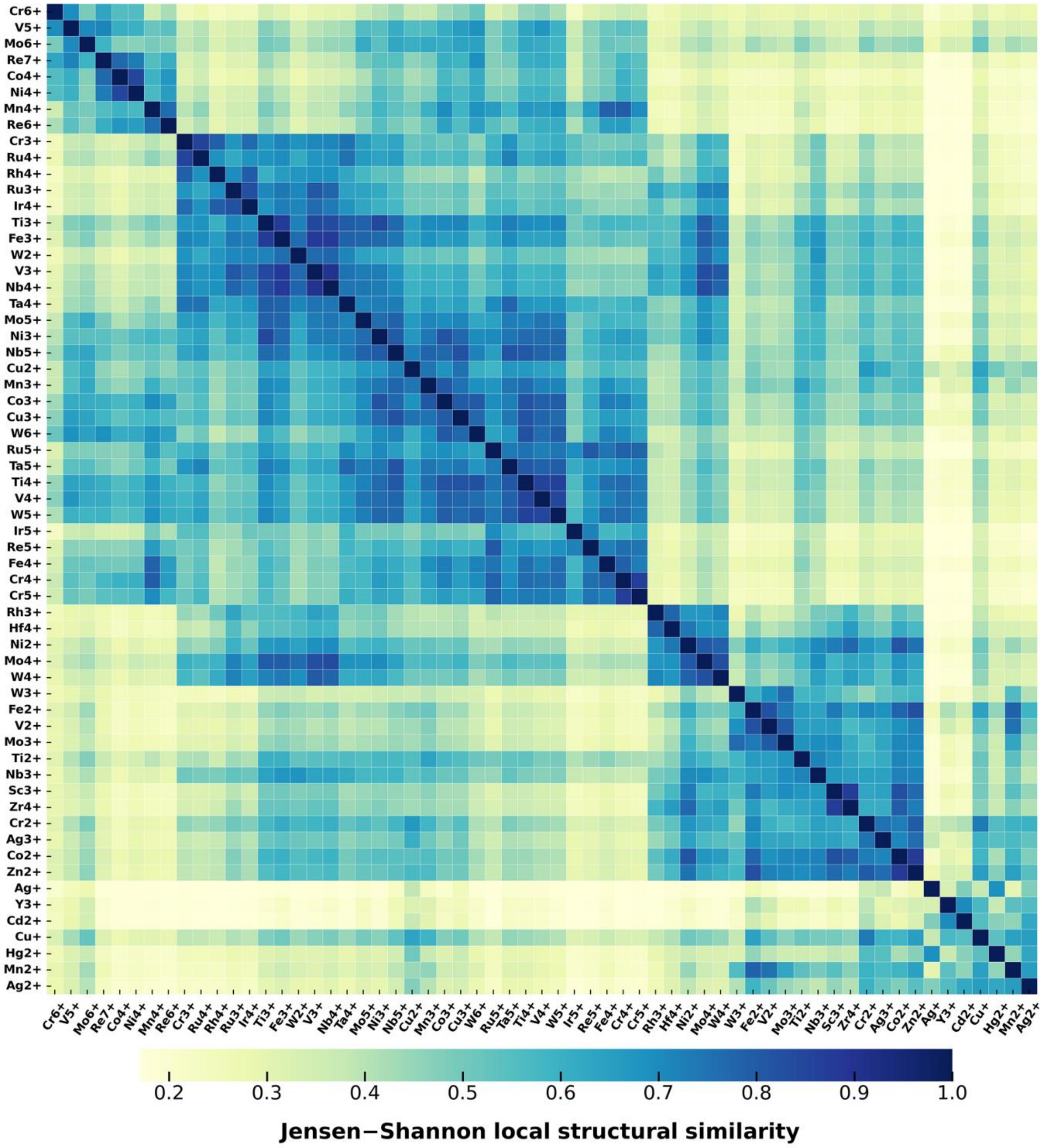


**Figure S3. The similarity of transition metal cations that derived from dataset of this work and Jensen−Shannon (JS) distance.**

We use Jensen−Shannon (JS) distance[1] $d_{\mathrm{JS}}(p_1 \parallel p_2)$ based on the Jensen−Shannon divergence[2] $D_{JS}(p_1 \parallel p_2)$ to recalculate similarity between the two distributions $p_1$ and $p_2$

$$d_{\mathrm{JS}}(p_1 \parallel p_2) = \sqrt{D_{\mathrm{JS}}(p_1 \parallel p_2)}$$

$$D_{\mathrm{JS}}(p_1 \parallel p_2) = \frac{1}{2} D_{\mathrm{KL}}(p_1 \parallel m) + \frac{1}{2} D_{\mathrm{KL}}(p_2 \parallel m)$$

where $m$ is the average distribution $\frac{p_1+p_2}{2}$ and $D_{KL}(q_1 \parallel q_2)$ is the Kullback-Leibler divergence of $q_2$ from $q_1$

$$D_{\mathrm{KL}}(q_1 \parallel q_2) = \sum_i q_1(i) \log_2 \frac{q_1(i)}{q_2(i)}$$

A value closer to 0 means more similarity, closer to 1 means less similarity, contrary to the definition of Overlap similarity in Figure 4a. For a more intuitive comparison, we define Jensen−Shannon (JS) similarity as

$$\mathrm{Similarity}_{\mathrm{JS}}\,(p_1 \parallel p_2) = 1 - d_{\mathrm{JS}}(p_1 \parallel p_2)$$

and use it to draw the similarity map. Compared to Overlap similarity in Figure 4a, the JS similarity is only a small systematic drift of those values, but the overall relative size remains the same.

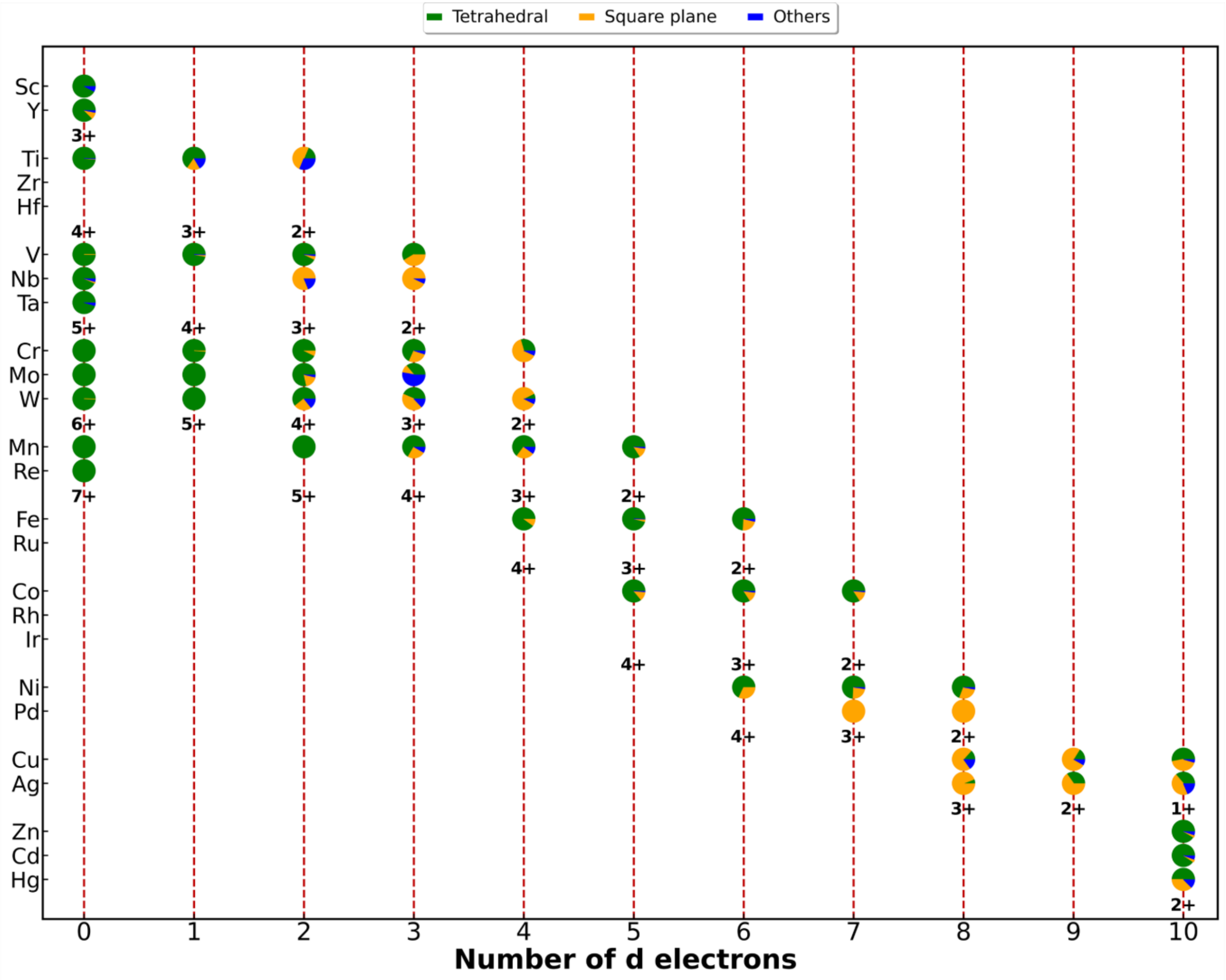


**Figure S4. The site geometry of the 4-coordination sites.** The 4-coordination sites can have multiple possible configurations, such as tetrahedral (green), square planar (yellow), and other shapes (blue). The analysis utilized the ChemEnv [3] module in the Pymatgen python library.

**Table S1. The average volume of the transition metal-oxygen octahedron in oxides.** The statistic is performed employing 5,930 compounds that exist in both the Materials Project (MP) and Inorganic Crystal Structure Database (ICSD). Considering that the MP data is obtained from a standardized density functional calculation at the GGA-PBE level with the Hubbard U correction and ICSD are mainly the experimental values, it can be concluded that the GGA-PBE normally overestimates the volume of cations by 3-6% and sometime ~9%. It equivalent to an overestimation of the bond length by 1-2% and sometimes 3%.

| Bond | MP volume ($Å^3$/atom) | ICSD volume ($Å^3$/atom) | MP-ICSD volume ratio |
|---|---|---|---|
| **Ag-O** | 14.744 | 13.905 | 1.062 |
| **Cd-O** | 13.693 | 12.899 | 1.062 |
| **Co-O** | 12.577 | 11.988 | 1.048 |
| **Cr-O** | 12.547 | 11.816 | 1.063 |
| **Cu-O** | 13.204 | 12.672 | 1.045 |
| **Fe-O** | 12.225 | 11.591 | 1.058 |
| **Hf-O** | 13.401 | 13.191 | 1.021 |
| **Hg-O** | 17.434 | 16.055 | 1.086 |
| **Ir-O** | 13.668 | 13.212 | 1.034 |
| **Mn-O** | 12.544 | 11.994 | 1.051 |
| **Mo-O** | 15.152 | 14.224 | 1.066 |
| **Nb-O** | 13.912 | 13.308 | 1.047 |
| **Ni-O** | 11.603 | 11.234 | 1.033 |
| **Pd-O** | 14.672 | 14.205 | 1.042 |
| **Re-O** | 14.463 | 13.690 | 1.056 |
| **Rh-O** | 12.470 | 11.792 | 1.069 |
| **Ru-O** | 13.582 | 13.050 | 1.041 |
| **Sc-O** | 12.758 | 12.250 | 1.041 |
| **Ta-O** | 13.717 | 13.168 | 1.044 |
| **Ti-O** | 12.608 | 12.259 | 1.034 |
| **V-O** | 13.408 | 12.685 | 1.057 |
| **W-O** | 14.317 | 13.453 | 1.068 |
| **Y-O** | 13.626 | 13.127 | 1.038 |
| **Zn-O** | 12.547 | 11.956 | 1.052 |
| **Zr-O** | 13.679 | 13.028 | 1.050 |

## Unsupervised Machine Learning Method

With the help of unsupervised machine learning methods, we can cluster TM cations into similar categories based on their local structural features without considering a priori knowledge. First, using local structure similarity defined by the bonds distribution overlap, we can get a similarity matrix from all TM cations. Then we used clustermap function in the seaborn package [4] to implement agglomerative hierarchical clustering of the similarity matrix for each cation category. The Unweighted Pair Group Method with Arithmetic Mean (UPGMA) method was used and the Euclidean distance metric was chosen to calculate the distance between each new cluster and the remaining cluster.

# References

[1] D. Waroquiers, X. Gonze, G.-M. Rignanese, C. Welker-Nieuwoudt, F. Rosowski, M. Göbel, S. Schenk, P. Degelmann, R. André, R. Glaum, *Chemistry of Materials* **2017**, *29* (19), 8346.

[2] D. M. Endres, J. E. Schindelin, *IEEE Transactions on Information theory* **2003**, *49* (7), 1858.

[3] D. Waroquiers, J. George, M. Horton, S. Schenk, K. A. Persson, G.-M. Rignanese, X. Gonze, G. Hautier, *Acta Crystallographica Section B: Structural Science, Crystal Engineering and Materials* **2020**, *76* (4).

[4] M. L. Waskom, *Journal of Open Source Software* **2021**, *6* (60), 3021.
